# The *FLC*-like gene *BvFL1* is not a major regulator of vernalization response in biennial beets

**DOI:** 10.3389/fpls.2014.00146

**Published:** 2014-04-14

**Authors:** Sebastian H. Vogt, Guy Weyens, Marc Lefèbvre, Bettina Bork, Axel Schechert, Andreas E. Müller

**Affiliations:** ^1^Plant Breeding Institute, Christian-Albrechts-University of KielKiel, Germany; ^2^SESVanderHave N.V.Tienen, Belgium; ^3^Strube Research GmbH & Co. KGSöllingen, Germany

**Keywords:** *Beta vulgaris*, bolting, *Flowering Locus C* (*FLC*), photoperiod, vernalization

## Abstract

Many plant species in temperate climate regions require vernalization over winter to initiate flowering. *Flowering Locus C* (*FLC*) and *FLC*-like genes are key regulators of vernalization requirement and growth habit in winter-annual and perennial *Brassicaceae*. In the biennial crop species *Beta vulgaris* ssp. *vulgaris* in the evolutionarily distant Caryophyllales clade of core eudicots growth habit and bolting time are controlled by the vernalization and photoperiod response gene *BTC1* and the downstream *BvFT1*-*BvFT2* module. *B. vulgaris* also contains a vernalization-responsive *FLC* homolog (*BvFL1*). Here, to further elucidate the regulation of vernalization response and growth habit in beet, we functionally characterized *BvFL1* by RNAi and over-expression in transgenic plants. *BvFL1* RNAi neither eliminated the requirement for vernalization of biennial beets nor had a major effect on bolting time after vernalization. Over-expression of *BvFL1* resulted in a moderate late-bolting phenotype, with bolting after vernalization being delayed by approximately 1 week. By contrast, RNAi-induced down-regulation of the *BvFT1*-*BvFT2* module led to a strong delay in bolting after vernalization by several weeks. The data demonstrate for the first time that an *FLC* homolog does not play a major role in the control of vernalization response in a dicot species outside the *Brassicaceae*.

## Introduction

Vernalization is the process by which the exposure of a plant to a prolonged period of cold temperatures over winter promotes the initiation of flowering. In temperate climate regions vernalization is an integral part of life cycle strategies as an evolutionary adaptation to changing seasons. In the annual dicotyledonous species *Arabidopsis thaliana* and its perennial relative *Arabis alpina*, the vernalization response is regulated by the MADS-box gene *Flowering Locus C* (*FLC*) and its ortholog *Perpetual Flowering 1* (*PEP1*), respectively (Michaels and Amasino, 1999; Sheldon et al., 1999; Wang et al., 2009; Zografos and Sung, 2012). By contrast, the vernalization response in monocotyledonous species like barley or wheat requires the *Vernalization 1-3* (*VRN1-3*) genes, with *VRN1* being the only MADS-box gene of these three (Yan et al., 2003, 2004, 2006). Recent studies in *Beta vulgaris*, which on an evolutionary scale is similarly distantly related to Arabidopsis (~120 million years of evolution) and the monocots (~140 million years; Chaw et al., 2004; Davies et al., 2004), revealed a new mode of life cycle control in dicotyledonous species. In *B. vulgaris*, the pseudo-response regulator (PRR) gene *Bolting Time Control 1* (*BTC1*) determines whether floral transition occurs in the first year of growth, as in annual accessions, or in the second year, as in biennials (Pin et al., 2012). *BTC1* mediates bolting and flowering by regulation of an antagonistic pair of *Flowering Locus T* (*FT*) homologs first described by Pin et al. (2010). Bienniality in beet derives from a recessive *BTC1* allele (*btc1*) with a reduced responsiveness to the floral inductive stimulus of long days and/or reduced activity of the BTC1 protein compared to annual beets. The perception of prolonged cold over winter after the first growing season restores the competence to bolt and flower in biennial beets. This process was suggested to involve up-regulation of *BTC1*, leading to suppression of the flowering repressor *BvFT1* and expression of the flowering activator *BvFT2*. Life cycle control by *BTC1* thus involves the integration of both photoperiod and vernalization signals. By contrast, the PRR genes in monocots such as *PPD1* in *Hordeum vulgare* or *SbPRR37* in *Sorghum bicolor* are only known to mediate photoperiod response, while a role in vernalization response or life cycle control has not been described (Turner et al., 2005; Murphy et al., 2011).

In Arabidopsis, FLC represses flowering by binding to *cis*-regulatory sequences in the floral integrator genes *FT* and *Suppressor of Overexpression of Constans 1* (*SOC1*) (Helliwell et al., 2006; Searle et al., 2006). During vernalization, *FLC* is down-regulated and the repressed state is epigenetically maintained after vernalization. The repression of *FLC* allows activation of *FT* under long-day conditions through the photoperiod pathway and its central regulator *Constans* (*CO*). FT protein expressed in the phloem companion cells of the leaves moves to the shoot apical meristem as part of the “florigen” signal and initiates flowering (Andrés and Coupland, 2012). Besides their highly conserved function as day length-induced floral activators, *FT* and *FT-like* genes also control other processes like stomatal opening in Arabidopsis or tuberization in potato (Pin and Nilsson, 2012).

A dose-dependent positive correlation between *FLC* expression and circadian period length was shown by using genotypes with different functional and non-functional allele compositions at *FLC* and the *FLC*-regulatory locus *FRIGIDA* (*FRI*) as well as a *35S::FLC* over-expressor line (Salathia et al., 2006). Furthermore, El-Assal et al. (2003) showed that *FLC* negatively and dose-dependently regulates expression of the photoreceptor gene *Cryptochrome 2* (*CRY2*). *CRY2* co-regulates circadian period length together with *CRY1* and tends to act as a negative regulator of period length (Devlin and Kay, 2000; Gould et al., 2013), suggesting that the *FLC*-induced increase in circadian period length may be mediated through cryptochromes. Vernalization resulted in a significant decrease in circadian period length, which was suggested to reduce the day length threshold required for photoperiodic induction of flowering and thus to accelerate flowering in spring (Yanovsky and Kay, 2002; Salathia et al., 2006). Finally, mutations in photoperiod pathway genes affected expression of *FLC*, providing further indication for the crosstalk between vernalization and photoperiod pathways (Rouse et al., 2002).

*FLC* and *FLC*-like genes belong to a major MADS-box gene clade that was recently shown to also include monocot genes (Ruelens et al., 2013). In dicots, *FLC*-like genes have been identified in two species outside the *Brassicaceae*, i.e., *B. vulgaris* in the Caryophyllales clade of core eudicots (Reeves et al., 2007) and the asterid species *Cichorium intybus* (Périlleux et al., 2013), which includes the biennial crop root chicory. Complementation analyses of *B. vulgaris FLC-LIKE 1* (*BvFL1*) and *C. intybus FLC-LIKE* (*CiFL1*) in Arabidopsis and down-regulation of *BvFL1* and *CiFL1* by vernalization in beet or chicory, respectively, suggested a conserved floral repressor function of these genes. However, instead of being epigenetically maintained in a transcriptionally silent state after vernalization, the expression of *BvFL1* and *CiFL1* after vernalization reverted to pre-vernalization levels (Reeves et al., 2007; Périlleux et al., 2013). Interestingly, the *FLC* ortholog *PEP1* in *A. alpina* also reverts to pre-vernalization expression levels after return to warm temperatures, which correlates with unstable histone modifications at the *PEP1* locus (Wang et al., 2009). Unstable repression of *PEP1* after vernalization was suggested to correlate with perennial life cycle strategies (Wang et al., 2009).

A more complex pattern of *BvFL1* regulation in beet emerged from a study of *BvFL1* expression in the shoot apical meristem (Trap-Gentil et al., 2011). According to this study, “bolting sensitive” biennial beet genotypes, which only require relatively short periods of vernalization for bolting to occur, are first down-regulated during vernalization, but up-regulated during a later stage of vernalization. The authors suggested that the early transient decrease in *BvFL1* expression during vernalization may account for the relatively high susceptibility to bolting in these genotypes. By contrast, “bolting resistant” biennial genotypes that require relatively long periods of vernalization exhibited a gradual increase in expression during vernalization. Furthermore, RNA methylation of *BvFL1* mRNA was detected in the shoot apical meristem of a bolting-resistant genotype after vernalization and was proposed to contribute to the control of vernalization response in sugar beet (Hébrard et al., 2013). However, a clear picture of the functional role of *BvFL1* in beet has not yet emerged, and a characterization of this gene's function through transgenic or mutational analyses in beet is still lacking.

Here, we further dissect the vernalization response in beet by over-expression and RNAi-mediated down-regulation of *BvFL1* and down-regulation of the *FT* homologs *BvFT1* and *BvFT2* in transgenic plants. Phenotypic analysis revealed a delay in bolting after vernalization by 1 week in transformants over-expressing *BvFL1*, while *BvFL1* RNAi neither had a major effect on bolting time after vernalization nor did it lead to bolting without vernalization. RNAi-induced concomitant down-regulation of the floral repressor *BvFT1* and the floral activator *BvFT2* resulted in a bolting delay by up to 7 weeks and a high percentage of non-bolting plants in a subset of transformation events. Taken together, our data support a dominant role of the *BvFT1-BvFT2* module in the control of vernalization response and show that, by contrast, *BvFL1* is not a major regulator of vernalization response in beet.

## Materials and methods

### Vector construction and plant transformation

For the *BvFL1* over-expression construct a 616 bp cDNA fragment covering the whole coding sequence of the splice variant *BvFL1_v3* (Reeves et al., 2007) was inserted downstream of a Cauliflower Mosaic Virus (CaMV) 35S promoter and the Tobacco Mosaic Virus (TMV) 5′UTR and upstream of an *Agrobacterium tumefaciens* nos 3′ terminator. In Arabidopsis, over-expression of *BvFL1_v3* caused the strongest delay in flowering among *BvFL1* splice variants (Reeves et al., 2007). RNAi vectors were constructed by insertion of a 332 bp fragment of the *BvFL1* 3′UTR or a 361 bp cDNA fragment spanning most of the phosphatidylethanolamine-binding protein (PEBP) domain of *BvFT1* (Pin et al., 2010), respectively, as inverted repeats between the regulatory elements described above. A 91 bp sugar beet intron sequence was used as spacer between the sense and antisense repeat units. The *phosphinothricin acetyl transferase* (*PAT*) gene was inserted downstream of the RNAi cassettes for selection of transgenic plants with glufosinate. The constructs were introduced into the biennial sugar beet genotype SES01 (SESVanderHave, Tienen, Belgium) by polyethylene glycol-mediated DNA transfer as described previously (Hall et al., 1996; Pin et al., 2012). Transgenic protoplasts, calli and regenerating plantlets were selected using glufosinate and transgene integration was confirmed by PCR. Low copy number (1–3 transgene copies) transformants were selected by quantitative PCR using TaqMan® assays (Life Technologies, Carlsbad, California, USA) and DNA gel blot analysis for the effector transgene and the *PAT* gene (Table 1). For DNA gel blot analysis, genomic DNA was digested with two different restriction enzymes, *Eco*RI and *Nco*I, separated by gel electrophoresis and transferred to Hybond™-N membranes (GE Healthcare, Little Chalfont, UK). Construct-specific probes were amplified from the corresponding plasmid DNA using primers 5′-CTATTTACAATTACACC ATGGCAGGCG and 5′-TGAACGATCGGGGAAATTCGAGCTCGG for analysis of *BvFL1* over-expression transformants, 5′-GGTTTTATATGTACTACTGTTGTAGCTG and 5′-TGAACGATCGGGGAAATTCGAGCTCGG for *BvFL1 RNAi* transformants, and 5′-GGTTTTATATGTACTACTGTTGTAGCTG and 5′-TGAACGATCGGGGAAATTCGAGCTCGG for *BvFT1-BvFT2 RNAi* transformants. A *PAT* gene-specific probe was amplified using primers 5′-AGATTAGGCCAGCTACAGCAGCTGATA and 5′-GCCTTGGAGGAGCTGGCAACTCAAAAT. Probes were radioactively labeled by random primer labeling (Feinberg and Vogelstein, 1983) using α-^32^P-dCTP and the large (Klenow) fragment of DNA polymerase I (Life Technologies, Carlsbad, California, USA). Copy number was determined as the number of discrete bands after hybridization. In cases where the number of detectable bands for a given transformant differed between the two enzymes, the detected range of copy numbers is given in Table 1. Transgenic and non-transgenic control plants were clonally multiplied *in vitro* and transferred to soil according to standard procedures (Hall et al., 1996).

**Table 1:** **Bolting time after vernalization in independent sugar beet transformants carrying *BvFL1* or *BvFT1-BvFT2* RNAi or over-expression constructs**.

**Effector construct type**	**Transgenic event number**	**Total number of vern. plants^a^**	**Number of bolting plants**	**DTB^b^ (mean ± SD^c^**	**Unpaired *t*-test value for DTB (probability^d^)**	**Number of non-bolting plants**	**Total number of non-vern. plants^e^**	***PAT* gene copy number^f^**	**Effector transgene copy number^f^**	**Target gene expression level**
***BvFL1***										
RNAi	019-07G	15	15	35.60 ± 3.20	6.85 (*p* < 0.0001)	0	n.a.	2	2	unchanged
RNAi	021-11G	15	15	37.00 ± 2.71	5.45 (*p* < 0.0001)	0	9	2	1	down
RNAi	021-12A	15	15	39.87 ± 4.84	1.16 (*p* = 0.2500)	0	n.a.	2	2	down
RNAi	021-12H	15	15	37.67 ± 4.13	3.72 (*p* = 0.0005)	0	n.a.	2	2	down
RNAi	022-10F	12	12	38.75 ± 3.24	2.68 (*p* = 0.0100)	0	n.a.	2	3	unchanged
RNAi	024-11E	20	20	36.55 ± 2.22	7.15 (*p* < 0.0001)	0	n.a.	1	1	down
RNAi	024-12E	20	20	44.15 ± 3.47	−4.06 (*p* = 0.0002)	0	6	1	1	down
Over-expression	016-05C	20	19	49.21 ± 6.31	−6.96 (*p* < 0.0001)	1	5	1	1	up
Over-expression	016-10A	15	14	41.07 ± 3.81	−0.05 (*p* = 0.9600)	1	8	2	3	up
Over-expression	017-06C	20	20	48.55 ± 5.62	−7.05 (*p* < 0.0001)	0	n.a.	2–3	2–3	up
Over-expression	017-07C	15	15	39.93 ± 4.19	1.20 (*p* = 0.2400)	0	n.a.	1	2	up
***BvFT1-BvFT2***										
RNAi	014-02G	20	18	48.83 ± 9.21	−4.79 (*p* < 0.0001)	2	n.a.	1	1	down
RNAi	014-07F	15	13	73.46 ± 18.37	−10.3 (*p* < 0.0001)	2	14	1–2	1	down
RNAi	014-08B	20	20	43.80 ± 5.78	−2.54 (*p* = 0.0140)	0	n.a.	3	2	unchanged
RNAi	018-06E	15	4	68.75 ± 5.80	−18.5 (*p* < 0.0001)	11	n.a.	1	2	down^g^
RNAi	018-09A	15	15	52.40 ± 4.99	−11.2 (*p* < 0.0001)	0	10	2	1	down
RNAi	019-01E	16	9	93.67 ± 36.73	−8.46 (*p* < 0.0001)	7	n.a.	3–4	3	down^g^
RNAi	020-01E	15	13	63.00 ± 8.49	−14.1 (*p* < 0.0001)	2	6	2	2	down
RNAi	018-12H	15	15	57.73 ± 7.64	−11.9 (*p* < 0.0001)	0	9	2	1	down
RNAi	020-01C	15	14	63.93 ± 25.27	−5.35 (*p* < 0.0001)	1	8	2	1	down
RNAi	020-05G	15	12	60.00 ± 15.04	−7.28 (*p* < 0.0001)	3	n.a.	2–3	2	down
***Non-transgenic control***										
n.a.	n.a.	37	37	41.03 ± 2.21	n.a.	0	n.a.	n.a.	n.a.	n.a.

a *Plants were generated by in-vitro multiplication (cloning) of primary transformants*.

b *Days to bolting after vernalization*.

c *Standard deviation of the mean*.

d *Probability that the DTB value is significantly different from that obtained using the non-transgenic control*.

e *For a subset of transformants, the number of plants indicated in this column was grown without vernalization in the greenhouse over spring and summer under optimal conditions for more than 6 months, but all of these plants failed to bolt*.

f *Copy numbers for the PAT gene and effector transgenes were determined by DNA gel blot analysis*.

g *High transgene expression*.

*n.a., not applicable*.

### Growth conditions and phenotyping

Transgenic plants and non-transgenic SES01 control plants were grown side-by-side in the greenhouse in early spring with supplementary lighting under 16 h light/8 h dark cycles. Vernalization and phenotyping was as described previously for *Bvbtc1 RNAi* transformants (Pin et al., 2012). In brief, vernalization was carried out in a climate chamber at 4°C and 16 h light for 3 months. To avoid devernalization several acclimation steps were performed over a period of 6 weeks during which the temperature was raised from 4 to 25°C during the light cycle and from 4 to 15°C during the dark cycle. Plants were phenotyped for the occurrence and time of bolting three times per week until 6 months after vernalization. Bolting time was defined as the number of days after the end of vernalization for a plant to reach a stem height of 5 cm. For each independent transgenic event 12–20 plants were phenotyped for bolting time. The non-transgenic control comprised 37 clones of the host genotype that was used for transformation. The Student's *t*-test was used for statistical analysis of phenotypic data. A subset of plants was not vernalized but instead continued to be grown in the greenhouse over spring and summer for more than 6 months under natural daylight conditions with supplementary lighting (16 h).

### Gene expression analysis

For each transgenic event and the non-transgenic control genotype, leaf samples of three clones each were harvested before vernalization 2 months after transfer to soil and again at the end of a 12 week vernalization period at Zeitgeber time (ZT) 6-8. For diurnal expression analysis, leaf samples of three individual clones of the *BvFL1* over-expressing transformant 016-05C were taken every 2 h over a period of 24 h 4 weeks after the end of vernalization. RNA extraction and cDNA synthesis were done for each of the three biological replicates (clones) separately and exactly as described for *Bvbtc1 RNAi* transformants (Pin et al., 2012). Primer annealing temperatures and elongation times are given in Table S1. Three technical replicates were performed for each RT-qPCR reaction. RT-qPCR was performed on a CFX96 Real-Time PCR detection system (Bio-Rad Laboratories, Hercules, California, USA) as described in Pin et al. (2012). To determine RT-qPCR efficiencies and serve as positive controls, the endogenous target transcript regions analyzed by RT-qPCR were cloned into the pGEM-T vector (Promega Corporation, Madison, Wisconsin, USA). All plant samples were assayed for expression of the respective RNAi or over-expression target gene and the housekeeping gene *BvGAPDH*, which was used as a reference gene for normalization. The comparative *C*_*T*_(2^−ΔΔ*CT*^) method was applied for analysis (Schmittgen and Livak, 2008).

## Results

### RNAi and over-expression of vernalization response genes in biennial beets

*BvFL1* and the *FT*-like gene pair *BvFT1-BvFT2* were analyzed by RNAi or over-expression in a biennial genetic background. Four to ten independent transformants were pre-selected for the presence of transgene inserts and low transgene copy numbers by PCR, TaqMan® assays and DNA gel blot analysis as described in Materials and Methods. Transformants with low copy numbers (1–3) were multiplied by clonal propagation and analyzed for changes in target gene expression and bolting time (Table 1; Table S1).

### BvFL1

**Figure 1:**
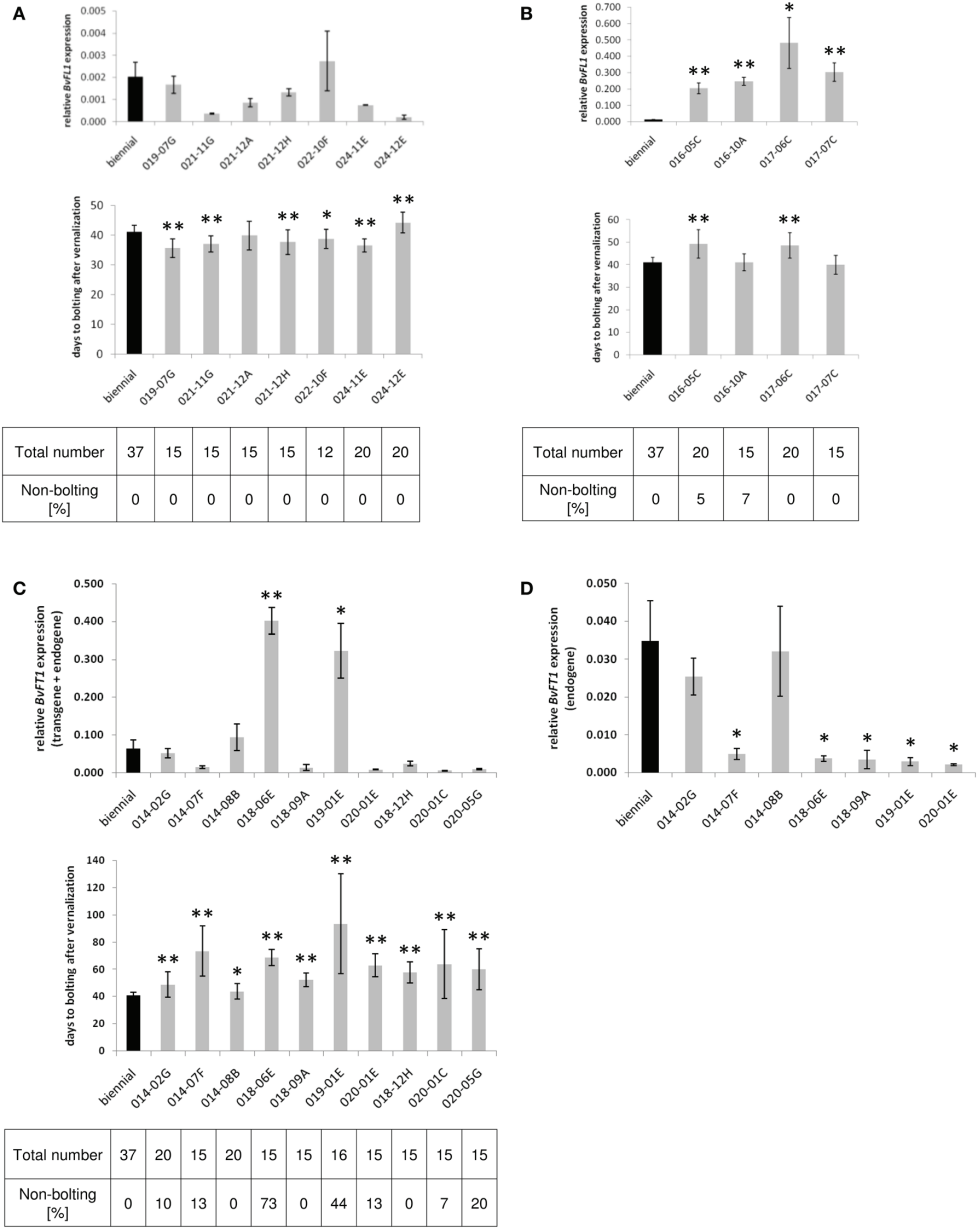
**Gene expression and bolting time phenotypes in *BvFL1 RNAi* (A), *BvFL1* over-expression (B), and *BvFT1-BvFT2 RNAi* transformants (C,D)**. Leaf samples of non-vernalized plants derived from independent sugar beet transformation events and the non-transgenic biennial control genotype were taken under long-day conditions at Zeitgeber time (ZT) 6-8. For each transgenic event, three clones were analyzed as biological replicates, and each RT-qPCR reaction was run in triplicate. Gene expression was normalized using the house-keeping gene *BvGAPDH* and the 2^−ΔΔ*CT*^ method (Schmittgen and Livak, 2008). Error bars represent mean ± SE of the mean. Expression of *BvFT1* in *BvFT1-BvFT2 RNAi* plants was determined with primers which co-amplify endogenous and transgenic *BvFT1* transcripts **(C)** and with primers which specifically amplify the endogenous *BvFT1* transcript **(D)**. Bolting time was measured in days to bolting after the end of vernalization. The mean of days to bolting and the SE of the mean are shown for plants which bolted within 6 months after the end of vernalization. Significant differences between expression levels in the transformants and the control plants and between bolting time are indicated by asterisks (^*^α = 0.05, ^**^α = 0.01 according to Student's *t*-test). The total number of plants per transgenic event and the percentage of plants which failed to bolt within this period are given in the tables below the bar graphs.

Down-regulation of *BvFL1* by RNAi to less than half of the expression level in the non-transgenic control plants was successful in four out of seven transgenic events (Figure 1A). Two of these events (021-11G, 024-12E) showed a reduction to less than 20% of the expression level in the control plants. Following vernalization, all *BvFL1 RNAi* transformants bolted (Figure 1A; Table 1). The mean days to bolting after the end of vernalization varied from 35.60 to 44.15 days, whereas bolting occurred on average 41.03 days after vernalization in the non-transgenic control plants. In one *BvFL1 RNAi* event, the mean days to bolting did not deviate significantly from the control plants. Five events bolted 2.28–5.43 days earlier and one event bolted 3.12 days later than the control plants. Of the four *BvFL1 RNAi* events in which *BvFL1* was down-regulated most, two (021-11G, 021-12H) bolted 3–4 days earlier than the control, one (024-12E) bolted 3 days later, and one (021-12A) did not deviate significantly from the control. Together, the data suggest a certain level of experimental noise but did not reveal a clear and consistent phenotypic effect of reduced *BvFL1* expression.

Of the four events derived from transformation with a *BvFL1* over-expression construct, all showed strong up-regulation of *BvFL1* expression (Figure 1B). Bolting time after vernalization varied from 39.93 to 49.21 days. In two events (016-05C, 017-06C), bolting was delayed by approximately 8 days, whereas in the two other events (016-10A, 017-07C) bolting time did not deviate significantly from the control (Table 1). Two events (016-05C, 016-10A) included one plant each which failed to bolt until the end of the experiment 6 months after the end of vernalization.

Of the two events with down-regulation of *BvFL1* to less than 20% of the control (021-11G, 024-12E) and two *BvFL1* over-expression events (016-05C, 016-10A), an additional 5–9 plants each were grown in parallel for more than 6 months over spring and summer under long-day conditions and without vernalization in the greenhouse, but none of these plants initiated bolting (Table 1).

### BvFT1-BvFT2

Out of ten transgenic events derived from transformation with an RNAi construct carrying part of the *BvFT1* cDNA, seven exhibited down-regulation of the *BvFT1* endogene (Figure 1C). A further analysis of several *BvFT1 RNAi* transformants revealed that not only *BvFT1* but also *BvFT2* was down-regulated in these plants, presumably due to RNAi off-target effects (see further below). Therefore, the RNAi transformants expose the effects of co-silencing of both constituents of the *BvFT1-BvFT2* module and will be referred to as *BvFT1-BvFT2 RNAi* events.

Bolting was delayed in all seven events and occurred approximately 8–32 days later than in the control plants (Figure 1C; Table 1). Besides a delay in bolting time, five of the seven *BvFT1-BvFT2 RNAi* events also included one to three non-bolting plants each among the 15–20 plants that were phenotyped for each of the *BvFT1-BvFT2 RNAi* events. An additional 47 plants (6–14 plants each of events 014-07F, 018-09A, 020-01E, 018-12H, 020-01C) were grown for more than 6 months without vernalization in the greenhouse and side-by-side with the *BvFL1* events mentioned above, but like these did not initiate bolting (Table 1).

Two of the transgenic events (018-06E and 019-01E) stood out in that they appeared to show over-expression of *BvFT1* rather than down-regulation (Figure 1C). The primer binding sites of the RT-qPCR assay for *BvFT1* expression were located within the segment of the cDNA that was used for construction of the inverted repeat cassette in the RNAi construct. Thus, both the endogenous *BvFT1* transcript and the transgene-derived transcript can be co-amplified, suggesting that transcription from the transgene may contribute to the observed high levels of transcript accumulation. To test this possibility, *BvFT1* expression was re-analyzed by RT-qPCR using endogene-specific primers (with binding sites outside the cDNA fragment used for RNAi transgene construction) in the two events in question as well as five of the events in which *BvFT1* expression was either down-regulated or largely unchanged. For the latter five events, this analysis confirmed the previous expression data. However, for the events in question the endogene-specific RT-qPCR now revealed clear down-regulation of the endogene (Figure 1D). Transcript accumulation in these two events was similarly low as in other transformants in which *BvFT1* was down-regulated (<20% of transcript accumulation in the control). Interestingly, the same two events also contained exceptionally high percentages of non-bolting plants (73% in 018-06E and 44% in 019-01E; Figure 1C; Table 1).

### Pre- and post-vernalization expression of floral regulators

**Figure 2:**
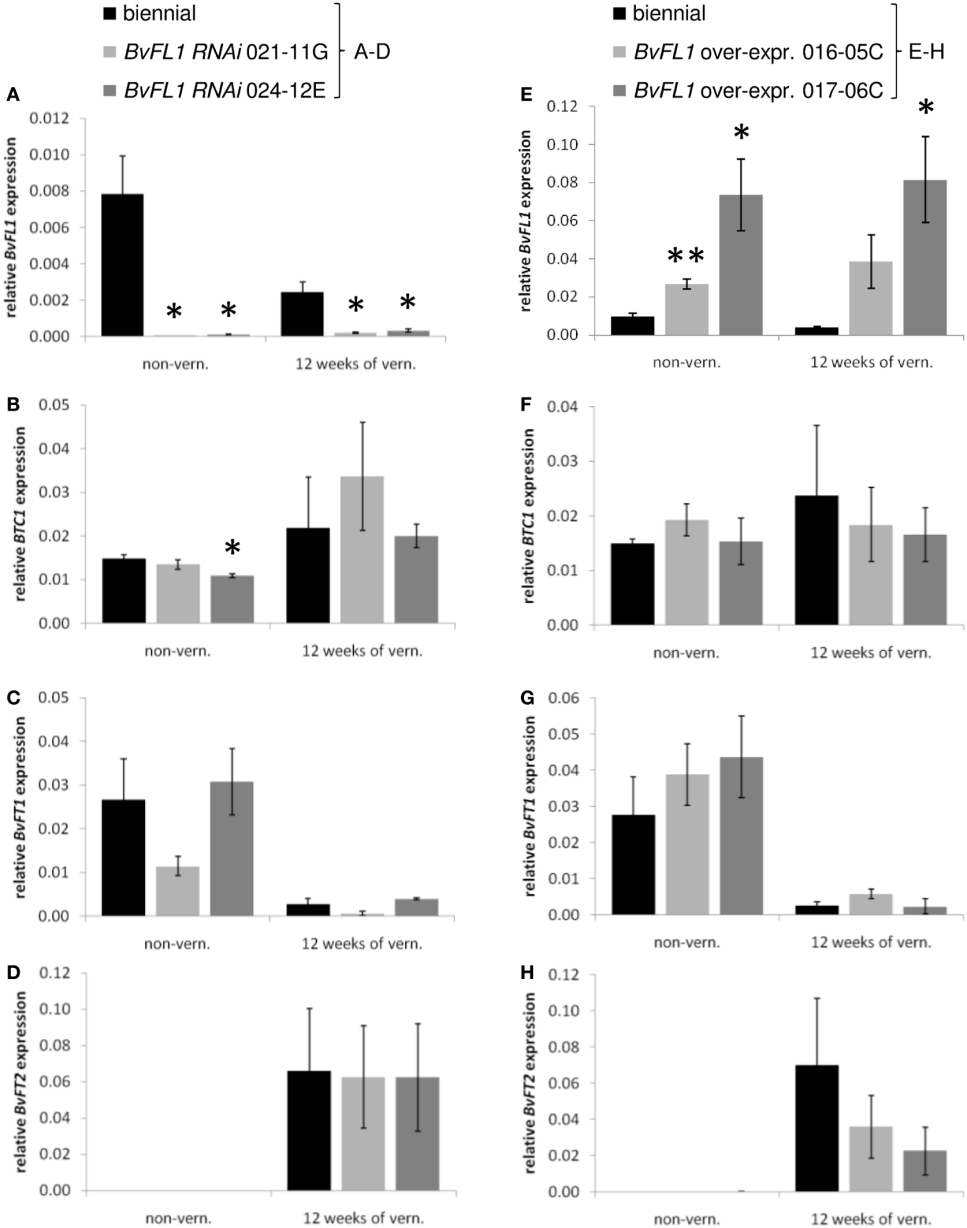
**Expression of floral regulators in *BvFL1 RNAi* (A–D) and *BvFL1* over-expression (E–H) transformants**. Expression of *BvFL1* **(A,E)** and the floral regulators *BTC1* **(B,F)**, *BvFT1* **(C,G)**, and *BvFT2* **(D,H)** was measured in leaves of non-vernalized plants and at the end of a 12-week vernalization period at ZT 6-8 under long-day conditions. Expression analysis, normalization, and statistical analysis was performed as described for Figure 1. Significant differences between expression levels in the transformants and the control plants are indicated by asterisks (^*^α = 0.05, ^**^α = 0.01 according to Student's *t*-test).

Two independent transgenic events each which showed either clear down- or up-regulation of the gene of interest were analyzed further. These events were assayed for target gene expression before vernalization and at the end of a 12-week vernalization period. Expression of *BvFL1* in the non-transgenic control plants was lower at the end of vernalization than before vernalization (Figure 2A). The strong down-regulation of *BvFL1* by RNAi in the transgenic events 021-11G and 024-12E when compared to the control plants was evident both before and at the end of vernalization. To test for possible regulatory effects on the three central flowering time control genes thus far identified in beet, the *BvFL1 RNAi* plants were analyzed for expression of *BTC1*, *BvFT1*, and *BvFT2* (Figures 2B–D). In the non-transgenic control plants, expression differences between samples harvested before and at the end of vernalization were largely consistent with previous reports (Pin et al., 2010, 2012), i.e., *BTC1* and *BvFT2* expression levels were higher at the end of vernalization than before vernalization, whereas *BvFT1* expression was strongly reduced at the end of vernalization. Down-regulation of *BvFL1* by RNAi did not result in consistent changes in expression of any of the central floral regulators.

In the two *35S::BvFL1* events which were further analyzed (016-05C and 017-06C) *BvFL1* was stably over-expressed both before and at the end of vernalization (Figure 2E). The difference in expression levels between the two events was in approximate accordance with the respective transgene copy numbers (1 in 016-05C and 2–3 in 017-06C; Table 1). *BTC1* expression did not appear to be majorly affected by *BvFL1* over-expression (Figure 2F). *BvFT1* expression before vernalization was slightly higher in the *35S::BvFL1* transformants than in the control plants but *BvFL1* over-expression did not prevent down-regulation of *BvFT1* by vernalization (Figure 2G). *BvFT2* expression was not detectable before vernalization in either the control or over-expression plants. *BvFT2* was expressed at the end of vernalization and was lower in the *BvFL1* transformants than in the controls (Figure 2H). None of the expression levels in the transformants deviated significantly from the control plants.

**Figure 3:**
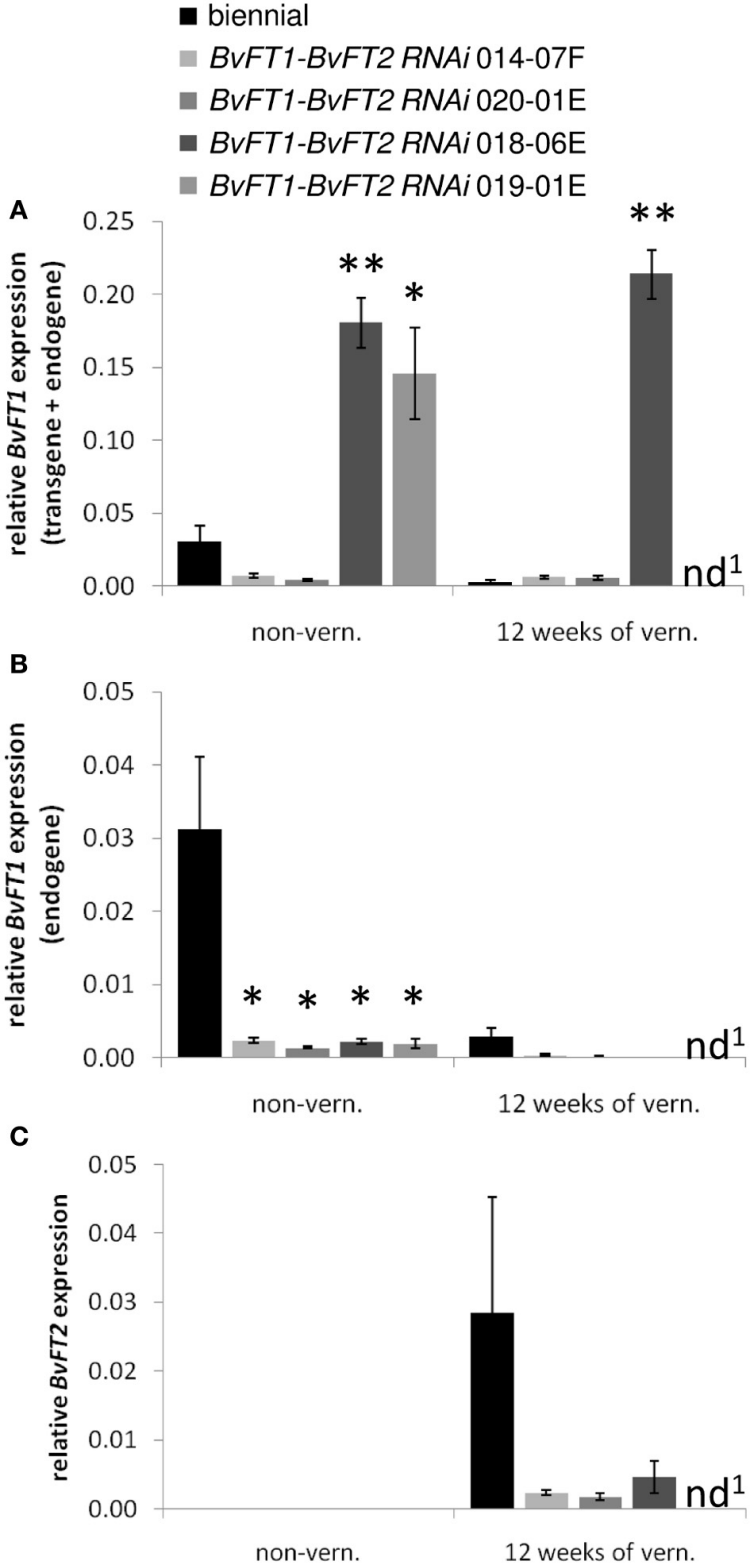
**Expression of *BvFT1* (A,B) and *BvFT2* (C) in *BvFT1-BvFT2 RNAi* transformants**. Expression was measured in leaves of non- vernalized plants and at the end of a 12-week vernalization period at ZT 6-8 under long-day conditions. *BvFT1* expression was determined either with primers which co-amplify endogenous and transgenic *BvFT1* transcripts **(A)** or with primers which specifically amplify the endogenous *BvFT1* transcript **(B)**. Expression analysis, normalization, and statistical analysis was performed as described for Figure 1. nd, not determined. Significant differences between expression levels in the transformants and the control plants are indicated by asterisks (^*^α = 0.05, ^**^α = 0.01 according to Student's *t*-test).

Expression analysis of *BvFT1-BvFT2 RNAi* plants before and at the end of vernalization showed down-regulation of both of the *FT* genes (Figure 3). Because *BvFT2* is only expressed after vernalization, down-regulation of this gene was only detectable in the post-vernalization samples (Figure 3C). As described above, *BvFT1-BvFT2 RNAi* transformants showed low accumulation of both endogene- and transgene-derived transcripts (including 014-07F and 020-01E; Figure 3) except for two events (018-06E, 019-01E) in which the transgene-derived transcripts accumulated to higher levels. The distinction between these two types of events was evident both before and at the end of vernalization.

### Diurnal expression profiles of floral regulators in *BvFL1* over-expression plants

Previous reports for Arabidopsis indicated a regulation of the circadian clock by *FLC* (Swarup et al., 1999; El-Assal et al., 2003; Salathia et al., 2006). Therefore, the late-bolting *BvFL1* over-expression event 016-05C, which carries a single copy of the transgene, was assayed for changes in the diurnal expression profiles of the beet homolog of the circadian clock gene *GIGANTEA* (*GI*) (Pin et al., 2012) and the photoperiod response gene *BTC1*. *BvFT1*, *BvFT2* and *BvLHP1*, a homolog of the vernalization pathway gene *LIKE HETEROCHROMATIN 1* in Arabidopsis (GenBank accession number KJ636469), were also included in the analysis. Diurnal expression was analyzed under long-day conditions (16 h light, 8 h darkness) 4 weeks after the end of vernalization.

**Figure 4:**
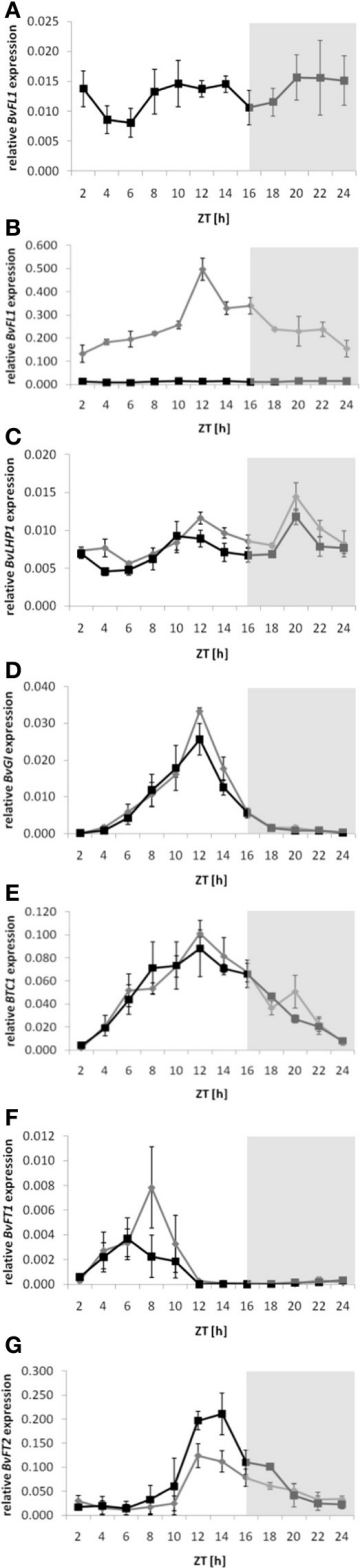
**Diurnal expression profiles of floral regulator genes or candidate genes in *BvFL1* over-expressing plants**. Expression in the *BvFL1* over-expressing transgenic event 016-05C (gray line and diamonds) and the biennial control genotype (black line and squares) was determined 4 weeks after the end of vernalization under long-day conditions. **(A,B)** *BvFL1*, **(C)** *BvLHP1*, **(D)** *BvGI*, **(E)** *BTC1*, **(F)** *BvFT1*, and **(G)** *BvFT2*. Expression analysis and normalization was performed as described for Figure 1.

In the non-transgenic control plants, *BvFL1* had two broad peaks of expression at mid-day to mid-afternoon and in the second half of the night until early morning (Figure 4A), indicating that *BvFL1* itself is diurnally regulated. Over-expression of *BvFL1* resulted in strongly increased transcript accumulation during the entire course of the day (Figure 4B). Transcript accumulation was not constant but peaked at ZT 12. Diurnal fluctuations of similar amplitude in expression from a CaMV 35S promoter in transgenic plants were observed before (Millar et al., 1992; Lu et al., 2011). *BvLHP1* transcript accumulation exhibited two peaks in the early afternoon (ZT 10) and in the middle of the night (ZT 20; Figure 4C). Over-expression of *BvFL1* correlated with a phase shift by approximately 2 h in *BvLHP1* expression during the light cycle compared to the control plants, resulting in a peak of expression at ZT 12. *BvLHP1* expression in the dark was in phase with the control. Expression of *BvGI* (Figure 4D) and *BTC1* (Figure 4E) was similar as reported previously (Pin et al., 2012). While expression of both genes reached its maximum around mid-afternoon, the peak of expression was broader for *BTC1*. Overall, the expression profiles of both *BvGI* and *BTC1* were similar in the *BvFL1* over-expressing plants and the control plants.

As reported before (Pin et al., 2010, 2012), the floral repressor *BvFT1* is only relatively weakly expressed after vernalization but exhibits detectable transcript accumulation in the morning hours, whereas expression of the floral activator *BvFT2* peaks around mid-afternoon. Expression of these genes in the control plants of the current study were in accordance with the previous reports (Figures 4F,G). However, the *BvFL1* over-expressing plants revealed the following deviations from the regular expression patterns in the control plants: (1) *BvFT1* expression showed a sharp peak of increased expression around ZT 8, and (2) *BvFT2* showed a reduction in gene expression in the afternoon and evening when compared to the control plants.

## Discussion

Recent studies have revealed a central role of the *B* locus gene *BTC1* and its downstream target gene *BvFT1* in vernalization response and bolting control in beet (Pin et al., 2010, 2012). Furthermore, in contrast to Arabidopsis, where vernalization requirement and growth habit is governed by natural variation at *FLC* or its upstream activator *FRI*, life cycle control in beet is determined by allelic variants of *BTC1*. Despite the apparent differences in the genetic make-up of the core regulatory modules in Arabidopsis and beet, an *FLC* homolog has been identified in beet (Reeves et al., 2007). Complementation analysis in an early-flowering *flc* mutant in Arabidopsis showed that the *FLC*-like gene *BvFL1* was able to rescue the wild-type phenotype, but the function of *BvFL1* has not yet been analyzed in beet. The current study aimed to address the functional role of *BvFL1* and possible regulatory interactions with *BTC1* and/or the *BvFT1-BvFT2* module by transgenic analyses in beet. The main findings are that (1) down-regulation of *BvFL1* neither affects bolting time majorly after vernalization nor enables bolting without vernalization, (2) over-expression of *BvFL1* is not sufficient to prevent bolting after vernalization but can result in a moderate delay of bolting, and (3) co-silencing of the *BvFT1-BvFT2* module in *BvFT1 RNAi* transformants leads to a stronger bolting delay than *BvFL1* over-expression and high percentages of non-bolting plants in some events.

The observed lack of a floral inductive effect in *BvFL1 RNAi* transformants stands in contrast to observations in Arabidopsis, where mutation or antisense-mediated down-regulation of *FLC* strongly accelerates flowering (Michaels and Amasino, 1999; Sheldon et al., 1999, 2000) and can eliminate the very late-flowering phenotype found in winter-annual (vernalization-responsive) accessions (Michaels and Amasino, 2001). This observation corroborates the notion from the work on *BTC1* and *BvFT1* that in beet a different regulatory switch has evolved for the control of growth habit, and shows for the first time that in beet altered regulation of the *FLC*-like gene is not sufficient to promote an early-bolting (annual) growth habit. The fact that *BvFL1 RNAi* transformants are responsive to vernalization further suggests that vernalization can promote bolting through a *BvFL1*-independent pathway. In Arabidopsis, despite the regulatory role of *FLC* in the vernalization pathway, *flc* null mutants are also vernalization-responsive, which suggested the presence of an *FLC*-independent vernalization response pathway also in this species (Michaels and Amasino, 2001). Later work implicated other MADS-box genes in *FLC*-independent regulation of vernalization response (Alexandre and Hennig, 2008). In beet, a *BvFL1*-independent vernalization response pathway is likely to involve at least in part the actions of *BTC1* and *BvFT1*.

While *BvFL1* may not have a key role in the regulation of vernalization requirement and response in beet, the moderate delay in bolting that was observed in transformants over-expressing *BvFL1* suggests that the gene has retained a functional role in the control of floral transition, and is consistent with the earlier complementation studies in Arabidopsis (Reeves et al., 2007). However, phenotypic effects of over-expression are not a definite proof of a gene's function in an endogenous biological process. For example, ectopic expression of the *A. thaliana* gene *FLC* in rice also delayed flowering despite the absence of *FLC*-like genes in rice (Tadege et al., 2003). With regard to growth habit, the biennial, vernalization-responsive sugar beet accession used in the current study is similar to winter-annual Arabidopsis accessions. Over-expression of *FLC* in winter-annual Arabidopsis accessions frequently resulted in transformants which completely failed to flower (Michaels and Amasino, 1999; Sheldon et al., 1999). In beet, however, complete suppression of bolting by *BvFL1* over-expression was not observed. The notion that *BvFL1* expression is not sufficient to prevent floral transition in beet also appears consistent with the previous finding that the temporary down-regulation of *BvFL1* during vernalization is reversed upon transfer to warmer temperatures (Reeves et al., 2007). The moderate phenotypic effects of altered *BvFL1* expression may suggest that the strong floral inhibitory effect of *BvFT1* (Pin et al., 2010) masks or overrides a possible contributory role of *BvFL1* in the repression of bolting.

In Arabidopsis, allelic variation at *FLC* was suggested to affect circadian period length (Swarup et al., 1999) and over-expression of *FLC* lengthened the circadian period by approximately 1 h (Salathia et al., 2006). Salathia et al. further argued that repression of *FLC* in response to vernalization and the resultant shorter circadian periods may reduce the critical daylength required for the photoperiod pathway to promote flowering, thus accelerating flowering in spring. In our study, expression of *BvFL1* showed diurnal oscillations both in the biennial control plants and the *BvFL1* over-expressing plants. While strong diurnal oscillations of *FLC* have not been reported in Arabidopsis (e.g., Fujiwara et al., 2010), a similar expression profile to that observed here for *BvFL1* was found by Lu et al. (2011), with peaks of *FLC* expression in the afternoon and at the end of the night. Among the putative clock-regulated genes analyzed in *BvFL1* over-expression plants, neither *BvGI* nor *BTC1* were majorly affected in their diurnal expression profiles. Expression of both of these genes and of *BvLHP1* was slightly elevated in the afternoon hours in the *BvFL1* over-expressing plants, but the differences were too subtle to be conclusive.

*FLC* inhibits floral transition at least in part by repression of *FT* in leaves, which involves a direct interaction of FLC protein with *FT* chromatin (Helliwell et al., 2006; Searle et al., 2006). Our data for beet tentatively suggest that *BvFL1* over-expression leads to a reduction of *BvFT2* expression, which is apparent both at the end of vernalization (Figure 2H) and in the diurnal expression profile 4 weeks after vernalization (Figure 4G). *BvFT2* expression in *BvFL1* over-expressing transformants rises more slowly in the mid-day hours and is reduced compared to the control plants during the afternoon and evening hours. This suggests that the observed bolting delay may also be mediated by negative regulation of *BvFT2* by *BvFL1* in beet. However, although *BvFT2* down-regulation is consistent in all samples and is apparent at multiple consecutive time points in the diurnal expression profile, the differences are not statistically significant in pairwise comparisons with the respective controls. Similarly, the analysis of *BvFT1* in *BvFL1* over-expression plants showed an increase in *BvFT1* expression which however was not statistically significant. Thus, it remains speculative whether changes in *BvFT1* and/or *BvFT2* expression mediate the observed bolting delay in *BvFL1* over-expression plants.

The current study also revealed phenotypic effects of co-silencing of *BvFT1* and *BvFT2*. *BvFT1* and *BvFT2* share 80% sequence identity at the nucleotide sequence level within the 361 bp region of the coding sequence that was used for RNAi vector construction, including a 23 bp tract of perfect sequence identity, suggesting that down-regulation of *BvFT2* is due to off-target effects. Down-regulation of *BvFT1* by RNAi had not been achieved previously (cf. Pin et al., 2010). Although down-regulation of *BvFT1* in the RNAi transformants investigated here was accompanied by down-regulation of *BvFT2*, the data provide new evidence for the critical role in bolting control of the *BvFT1-BvFT2* module in beet, and show that the concomitant down-regulation of both activities inhibits rather than promotes bolting. The data also suggest that *BvFT1* expression before vernalization as it is typical for biennial beets is not necessary for pre-vernalization development and that the main function of *BvFT1* is its role in the control of vernalization response. This notion is consistent with the apparent lack of *BvFT1* expression in annual beets throughout development (under long-day conditions; Pin et al., 2010). All *BvFL1 RNAi*, *BvFL1* over-expression and *BvFT1-BvFT2 RNAi* transformants investigated here were grown and analyzed side-by-side with each other as well as with the *btc1 RNAi* transformants described by Pin et al. (2012), thus facilitating a comparative view. The strong phenotypic effect of altered regulation of the *BvFT1-BvFT2* module in *BvFT1 RNAi* transformants when compared to *BvFL1* RNAi or over-expression point at the predominant role of the *FT* genes in bolting control in beet. Finally, it is also interesting to note that among all the *BvFL1*, *BvFT1-BvFT2*, and *btc1* RNAi transformants, it was clearly the *btc1 RNAi* transformants which showed the strongest suppression of bolting, with multiple transgenic events in which bolting was completely suppressed until the end of the experiment 6 months after the end of vernalization (Pin et al., 2012).

Perhaps noteworthily, the strongest inhibitory effect on bolting was found in two transgenic events (018-06E and 019-01E) in which the *BvFT1* transgene was highly expressed despite a strong reduction in accumulation of the endogenous *BvFT1* transcript. A large number of plants derived from these events failed to bolt after vernalization [11 out of 15 plants (73%) and 7 out of 16 plants (44%), respectively], while the remaining plants of these events bolted very late and showed a stunted phenotype similar to *btc1 RNAi* transformants (Pin et al., 2012). The concomitant accumulation of the transgene transcript and silencing of the endogenous transcript may suggest that in these transformants transgenic and endogenous transcripts are not co-suppressed, but that the transgene transcript may trigger RNAi of the endogenous transcript without itself being a target of (efficient) RNAi-mediated transcript degradation. Because the two transformants carry multiple copies of the transgene it is conceivable that at least one of these copies carries the complete *BvFT1* inverted repeat cassette and effects RNAi, whereas another copy may have integrated only partially and escaped silencing. The cDNA fragment used for RNAi transgene construction spans ~67% of the full-length coding sequence and covers 88% of the central PEBP domain, including the functionally important amino acids in the fourth exon (Pin et al., 2010). The putative translation product, starting with the first in-frame ATG codon downstream of the 35S promoter, is predicted to contain 92 amino acids (~51%) of the full-length protein and ~67% of the PEBP domain. Thus, it is conceivable that expression of a partial BvFT1 protein at least contributes to the particularly strong inhibition of bolting observed in these transformants. In this scenario, the protein sequence outside the 92 amino acid region would appear to be dispensable for repression of bolting by BvFT1.

In conclusion, our data show that *BvFL1* is not a major regulator of vernalization response in beet. A comparison with phenotypic data from *BvFT1-BvFT2 RNAi* plants and our previously described *btc1 RNAi* transformants further suggests that in beet the *BvFT1-BvFT2* module and its upstream regulator *BTC1* have evolved a more dominant role in the control of vernalization reponse and bolting time. Future comparative studies between both species may help to uncouple the contributions of *FLC* and *FLC*-like genes to floral regulation through direct effects on *FT* genes or upstream interactions between vernalization- and photoperiod responsive flowering time control mechanisms. From an evolutionary perspective, knowledge of conservation and divergence of floral control mechanisms between model species and the phylogenetically distant dicot species *B. vulgaris* is casting an increasingly interesting light on one of the best studied developmental processes in plants.

## Author contributions

Sebastian H. Vogt designed and performed experiments and wrote the manuscript. Guy Weyens and Bettina Bork designed and performed experiments. Marc Lefèbvre and Axel Schechert designed and supervised experiments. Andreas E. Müller designed and supervised the project and wrote the manuscript.

### Conflict of interest statement

The authors declare that the research was conducted in the absence of any commercial or financial relationships that could be construed as a potential conflict of interest.
