# Supplementary material for: The FLC-like gene BvFL1 is not a major regulator of vernalization response in biennial beets
Source: Front Plant Sci. 2014 Apr 14;5:146. doi: 10.3389/fpls.2014.00146 (PMC3995057; doi:10.3389/fpls.2014.00146)
Supplement: Supplementary file 1 [file DataSheet1.PDF]

Supplementary Table 1. Primer sequences and PCR conditions for vector construction and RT-qPCR assays.

Gene	Reference	Primer	Primer sequence 5´->3´	Annealing conditions	Purpose
BTC1	Pin *et al.*, 2012	B580	GTGAAAGCTGTGTAAGGAATGG	61.0°C, 30“	RT-qPCR
		B581	AAGTTCCTGCATGGATCCAG		
BvFL1	Reeves *et al.*, 2007	B591	CATTTTCAAAGCGGCGTTCTG	57.0°C, 30“	RT-qPCR
		B592	ATTGAAGCTTTCCCGTCTGC		
BvFT1	Pin *et al.*, 2010	SELA4672	GCATCATTTGGAGAAGAGATTGTTTAC	64.5°C, 30“	RT-qPCR
		SELA4673	GGCGTTGTTGTGGAGCATTTA		
		B598	GTAACCCGCATCAAAGAGAG	64.5°C, 30“	RT-qPCR
		B594	CTAAAACCTCCTTCCACCAC		
BvFT2	Pin *et al.*, 2010	B584	GAGCCCAAGTAATCCACACTTG	64.5°C, 30“	RT-qPCR
		B585	GTGTTGAAGTTTTGACGCCAC		
BvGI	Pin *et al.*, 2012	BvGI-F	CACCATCATGGGCTCCAGA	60.0°C, 30“	RT-qPCR
		BvGI-R	AGTTTCTTGGCAATCTCATCCC		
BvLHP1	G. Schulze-Buxloh and A. Müller, unpublished	B609	CTGATGGGAAGGAAGTTATGG	64.5°C, 30“	RT-qPCR
		B610	GGACTATATCGCAGATGCTG		
BvFL1	Reeves *et al.*, 2007	B180	CATAAGGCGCGCCCCATGGGCTGTAAGAGTCGGCATTGAG	57°C, 25“	vector construction (RNAi)
		B181	CGATACATTTAAATGGTACCCCCAAGATAACTGAAGACTGAACC		
		B182	CATAACCATGGGGTATTTGGATAAGGATGG	57°C, 40“	vector construction (over-expression)
		B183	GATACGAGCTCTTACAGCAGTTACCTCAGC		
BvFT1	Pin *et al.*, 2010	B092	CATAAGGCGCGCCCCATGGGGAGATTTTAGGCCATCACAAG	57°C, 25“	vector construction (RNAi)
		B093	CGATACATTTAAATGGTACCATACAGCAGCAACAGGCAAG		
BvGAPDH	Reeves *et al.*, 2007	B582	GCTTTGAACGACCACTTCGC	61.0°C, 30“	RT-qPCR
		B583	ACGCCGAGAGCAACTTGAAC		
